# Personality—A Review
1*Personality*. By R. G. Gordon, M.D., B.Sc., M.R.C.P. Ed., London: Kegan Paul, Trench, Trübner & Co. Ltd. 1926.


**Published:** 1926

**Authors:** C. Lloyd Morgan


### PERSONALITY[1]—A REVIEW.

By Professor C. Lloyd Morgan, F.R.S.

"Why," asks Dr. Gordon in his Preface, "should a practising physician write on personality?" Why not, if, as a practising physician who is also a man of wide interests, he find in personality a central thought through which his outlook on human affairs is rendered coherent? It may be that the concept of personality Dr. Gordon develops is rather different from that which others have sought to develop. So much the better. This implies that he has a distinctive point of view which may be compared and correlated with other points of view.

There is presented, then, in this book a concept of personality. What is it? For a full answer to this question the whole work must be read with the care it deserves. But in the Introduction Dr. Gordon gives, first, a broad characterisation, and, later, a somewhat more specific definition—so far as a definition is possible—of the concept of personality he has been led to find helpful. It is not lacking in comprehensiveness. To get an adequate concept of a man's personality "we must," he says, "describe his parentage and race, his bodily structure, his intellectual attainments, his emotional reactions, his practical achievement, and all the slings and arrows of outrageous fortune that have fallen upon him from birth to death" (p. 6). That is a fairly large order. It states an unattainable ideal. Following it up in such detail as is possible, one can descry no end to the bewildering multiplicity of the seemingly heterogeneous events which constitute what one may call the component "stuff" of personality. But all these events—literally all without exception—ideally go together as one whole with substantial unity. It is this unity which we recognise as essential to personality. Hence, notwithstanding the baffling multiplicity disclosed by analysis, that on which Dr. Gordon bids us lay stress is the synthetic unity of this or that person in whose act and thought is embodied and enminded the reality to which our concept of personality affords an avenue of approach.

More than this. Dr. Gordon is whole-hearted in his acceptance of that which has been called "emergence." Stated generally and in abstract terms this means that, given a number of items of "stuff," say *a*, *b* and *c*, which go together

[1] *Personality.* By R. G. Gordon, M.D., B.Sc., M.R.C.P. Ed., London: Kegan Paul, Trench, Trübner & Co. Ltd. 1926.

in some assignable mode of relatedness, the synthetic whole (*abc*) has a quality or character of its own which is not the algebraic sum of the characters of *a*, *b* and *c* taken severally. Similarly such wholes of the first order may combine to form more complex wholes of the second order and so on in an ascending order of emergents. The emergent entity always has a quality or qualities other than those of the components which analysis may disclose. In many ways and in differing contexts Dr. Gordon applies this principle of interpretation. Personality is a crowning outcome of emergent evolution. It may "therefore be defined as the emergent synthesis of the bodily and mental attributes of the individual in relation to the environment" (p. 13). But its distinctive quality is indefinable. One must *be* a person to realise personality.

Since the synthesis is "bodily and mental" the relationship of body and mind is discussed in the second chapter. It must here suffice to say that the hypothesis of correlation or concomitance is accepted and sundry implications of alternative hypotheses such as animism or vitalism are explicitly rejected in subsequent discussion. Dr. Gordon's contention is that psychology, as science of mind, and physiology, as science of body, deal not with different sets of natural events but with differing kinds of relatedness (under different "laws") in the same organism. This entails a distinction between what one may speak of as two concurrent stories of these events, a physiological story to be told in terms of physical influence, action and reaction, under the concepts of stimulus and response, and a psychological story to be told in other terms —I should say in terms of mental reference to something objective and in terms of "enjoyment" in experiencing.

It is Dr. Gordon's aim to aid us in realising that there are always these two stories, and that though, as stories, they are distinguishable, they deal with one set of events in one organism of emergent person, as he uses this word. There are those, however, who prefer to reserve the word "person" (and the word "personality") for use in the story of mind, as such, and even here only when reflective thought is emergent. For them man, and man only, attains the status of person in virtue of his distinctively mental relatedness to other men who have attained a like status. They would also in some way distinguish personality from individuality, whereas Dr. Gordon often uses these two words as synonymous. They might, perhaps, have something more to say on the subtle relations of the concepts of person, individual, subject, ego, and self—

distinguishing, as I have elsewhere sought to distinguish, the self of enjoyment," say, in the infant, from the "self for contemplation" in the adult; and so on. This brings us back to what Dr. Gordon himself says in the Preface, that his concept of personality is rather different from that to which some others have been led. We must thank him for presenting a definite thesis, and not find fault with him if his concept is "rather different from" ours. And we must realise that his concept does lay due stress on the substantial unity which is common to all body-mind events alike in their physical and in their mental kinds of relatedness.

In any case his contention, though not under this phraseology, is that there are always the two stories. If, on the one hand, we tell a neurone-story, or a story of endocrine influence—and these stories are quite admirably summarised with due critical caution—let us not forget that there is an accompanying mind-story which tells of a kind of relatedness that is inalienably present from first to last, and counts more and more as higher levels of emergence are reached. If, on the other hand, we learn to tell the mind-story from its first to its last chapter, let us not forget that there is an accompanying body-story with physiological relatedness which is always inalienably compresent, and counts no less when the very highest levels of emergence are reached than in the more simple conditioned responses. It is the substantial unity in the set of events of which both stories may be told that is for Dr. Gordon the hall-mark of that which ultimately emerges as human personality.

Many readers will turn with special interest to the chapters which deal with these contributions to the study of personality which we owe to Freud, Jung, Adler and Kempf. They bring one into touch with what the writers themselves have taught, and not merely with second-hand, and too often second-rate, glosses. They are discussed with helpful touches of criticism by one who not only watches but plays the difficult psycho-analytic game, as cricketer and not merely as onlooker. They will amply repay careful perusal. But I want something more; and I know of no one who is better equipped than Dr. Gordon to supply what I, and I think many others, want. Most of us are prepared to welcome every fact in normal or so-called abnormal experience which is disclosed by any accredited method of analysis, psycho-analytic or other. But we find that these facts are too often interpreted in terms of drama, that is, in terms of personified entities, and not in terms of science, that is, in terms of mental relatedness, as such, under objective reference which may be correlated with physiological relatedness under the give and take of physical influence which, at the emergent level of life, takes the form of external stimulation or internal excitation with consequent response. What we want, then, is a thorough overhauling of all those current expressions which imply, or seem to imply, dramatic personification, and their translation into expressions which imply no more than the form which mental reference to something objective, actual or imaginative, then and there assumes. Dr. Gordon in some measure helps us to do so. But much more remains for him, or someone else, to do. If it could only be done and well done, this would herald a new era of scientific advance.

Let me illustrate what I mean by an analogy based on the principle of emergence. Take the molecularity of the molecule as analogous to the personality of the person. Under analysis the molecule is resolved into constituent atoms, which, as such, give no indication of the emergent character of molecularity. In brief, the constituent atoms are not sub-molecules. Now in the analysis of the person we may find, let us say, that which in dramatic terms may be spoken of as the good knight Censor holding in check the ugly demon Lust who assumes all sorts of weird disguises in order to escape the good knight's vigilance. No doubt for dramatic treatment these may be dealt with as sub-persons, who play their several parts in the comedy or tragedy of daily life. But they may have no more of the emergent character of personality than the analogous atoms have of the emergent character of molecularity.

Some such overhauling of current modes of dramatic expression as I have suggested would throw further light on the matters which Dr. Gordon discusses in chapters on "The Neurotic" and "The Delinquent," and those on "Dissociation and Retardation in Personality."

A final chapter deals with "The Spiritual Aspect of Personality" in relation to a comprehensive philosophy.

A work of this kind may be reviewed in the light of one's agreement or disagreement with the conclusions that are reached. A better principle of appraisal is based on assigning the work to one of two classes—the small class of works that count, and the far larger class of those that are negligible. Without hesitation I assign to Dr. Gordon's *Personality* a place among those that count.

